# Research on the Mechanism of Qushi Huayu Decoction in the Intervention of Nonalcoholic Fatty Liver Disease Based on Network Pharmacology and Molecular Docking Technology

**DOI:** 10.1155/2020/1704960

**Published:** 2020-11-04

**Authors:** Shan-shan Gao, Ji-jia Sun, Xin Wang, Yi-yang Hu, Qin Feng, Xiao-jun Gou

**Affiliations:** ^1^School of Pharmacy, Shaanxi University of Traditional Chinese Medicine, Xianyang, Shaanxi 712046, China; ^2^Department of Mathematics and Physics, Pharmacy School, Shanghai University of Traditional Chinese Medicine, Shanghai 201203, China; ^3^Institute of Liver Disease, Shuguang Hospital, Shanghai University of Traditional Chinese Medicine, Shanghai 201203, China; ^4^Central Laboratory, Baoshan District Hospital of Integrated Traditional Chinese and Western Medicine of Shanghai, Shanghai University of Traditional Chinese Medicine, Shanghai 201999, China

## Abstract

**Objective:**

To use network pharmacology and molecular docking technology in predicting the main active ingredients and targets of Qushi Huayu Decoction (QHD) treatment in Nonalcoholic Fatty Liver Disease (NAFLD) and explore the potential mechanisms of its multi-component-multi-target-multi-pathway.

**Materials and Methods:**

The main chemical components of QHD were searched using traditional Chinese medicine system pharmacology technology platform (TCMSP) and PubChem database. The main chemical components of the prescription were ADMET screened by the ACD/Labs software. The main active ingredient was screened by 60% oral bioavailability, and 60% of “bad” ingredients were removed from the drug-like group. Swiss Target Prediction, the SEA, and HitPick systems were sequentially used to search for the target of each active ingredient, and a network map of the QHD's target of the active ingredient was constructed. Genome annotation database platforms (GeneCards, OMIM, and DisGeNET) were used to predict action targets related to fatty liver disease. “Drug-Disease-Target” network diagram could be visualized with the help of Cytoscape (3.7.1) software. UniProt and STRING database platforms were used to build a protein interaction network. The KEGG signal pathway and DAVID platform were analyzed for biological process enrichment.

**Results:**

A total of 128 active ingredients and 275 corresponding targets in QHD were discovered through screening. 55 key target targets and 27 important signaling pathways were screened, such as the cancer pathway, P13K-AKT signaling pathway, PPAR signaling pathway, and other related signaling pathways.

**Conclusions:**

The present study revealed the material basis of QHD and discussed the pharmacological mechanism of QHD in fatty liver, thus providing a scientific basis for the clinical application and experimental research of QHD in the future.

## 1. Introduction

Over recent years, the incidence of fatty liver has been increasing, especially among the younger population [1]. Fatty liver disease is pathologically characterized by hepatocyte steatosis and fat accumulation caused by different factors. It can be divided into alcoholic fatty liver and Nonalcoholic Fatty Liver Disease (NAFLD) [2]. NAFLD refers to the clinical-pathological syndrome of hepatic steatosis caused by excessive deposition of triglyceride- (TG-) based lipids in hepatocytes, excluding excessive drinking history and obvious risk factors for liver damage. The pathogenesis of NAFLD has not been fully elucidated and is currently believed to be related to genetics, associated diseases, lifestyle, environment, and other multiple gene factors [3]. NAFLD is considered a more extensive liver manifestation of potential metabolic dysfunction and is closely related to many metabolic risk factors, including insulin resistance, dyslipidemia, cardiovascular disease, and the most severe obesity [4].

Inflammation has an essential role in the pathological process. NAFLD has become one of the risk factors affecting liver health. It has also become the second largest liver disease in China after viral hepatitis. Therefore, NAFLD has attracted increased attention; nonetheless, there is still no effective drug for the treatment of NAFLD. The commonly used drugs for the treatment of NAFLD are statins, which have a poor curative effect and may increase the risk of liver burden [5]. Traditional Chinese medicine (TCM) believes that the disease is mostly caused by poor diet, excessive obesity, emotional disorders, and other causes, such as the unhealthy spleen, qi deficiency related to the kidneys, damp-heat, turbid phlegm, blood stasis, fat accumulation, blocked circulation of the liver, and portosystemic collaterals [6]. Based on syndrome differentiation and treatment, combined with the application of soothing liver and dredging collaterals, removing blood stasis and turbidity, invigorating spleen and expelling phlegm, invigorating qi and activating blood circulation, clearing away heat and soothing liver, eliminating food, and so on, TCM treatment can achieve a good therapeutic effect, which has certain advantages in the treatment of NAFLD [7, 8].

QHD is a traditional Chinese medicine prescription for the treatment of NAFLD, which includes the following herbaceous plants: *Polygonum cuspidatum* (*Polygonum cuspidatum Sieb*, *HZ*), *Yinchen* (*Artemisia scoparia Waldst*, *YC*), *gardenia* (*Gardenia jasminoides Ellis*, *ZZ*), *turmeric* (*Curcuma longa L*., *JH*), and *Tianjihuang* (*Hypericum japonicum Thunb*, *TJH*) [9]. Previous studies have confirmed the effect of QHD on experimental NAFLD. Network pharmacology is a new subject based on the theory of system biology, which analyzes the biological system and selects specific signal nodes (nodes) for a multitarget drug molecular design. Network pharmacology emphasizes multi-component-multi-target-multi-pathway regulation of the signal pathway. From the perspective of molecular biology, the active components in TCM are linked with target genes [10]. In this study, we used the related target proteins of NAFLD as reference materials, involving NF-*α* [11], IL-6, and PPAR-*α* [12], and used network pharmacology and molecular docking technology to explore the mechanism of QHD in the treatment of NAFLD, so as to provide a scientific basis for clinical application.

## 2. Materials and Methods

### 2.1. QHD Component Collection and ADMET Screening

The main active components in the prescription of QHD (*Polygonum cuspidatum*, *Herba Yinchen*, *turmeric*, *Gardenia jasminoides*, and *Tianjihuang*) were retrieved from the TCMSP database. The chemical composition of QHD was established, and it was as follows: *Polygonum cuspidatum* had 52 components, *turmeric* had 39 components, *Yinchen* had 49 components, gardenia had 85 components, and *Tianjihuang* had 38 components. All ingredients were confirmed through PubChem (http://pubchem.ncbi.nih.gov) database. In addition, the PubChem ID and canonical SMILES of each component were collected.

The ACD/Labs software was used to perform ADMET screening of the main chemical components of QHD. The effective components were screened based on six indicators including Lipinski, solubility, bioavailability, metabolic stability, Ames, and hERG.

Among these, Lipinski's rule of 5 was the basic criterion for screening drug-like molecules. In the process of screening, the “bad” component was removed. The “solubility” was the index of compound solubility, and the “highly insoluble” component was removed during screening. Bioavailability was the prediction of oral utilization, and the compounds with a value higher than 60% (double reference value mg = 50) were taken as the effective components. Metabolic stability is an indicator used to measure the stability of drug metabolism. In ADMET evaluation, “unstable in HLM” was removed. Ames and hERG are, respectively, genotoxicity and cardiotoxicity indicators, and the components with clear toxicity were removed after screening.

### 2.2. Target Prediction and Recognition of Active Components

First of all, the canonical SMILES data on QHD, SEA (http://sea.bkslab.org/), HitPick (http://mips.helmholtz-muenchen.de/proj/hitpick), Swiss Target Prediction, and other databases were used to predict the main effective chemical components of QHD. Among them, the score value max was selected from the SEA database. The prediction results of TC > 0.6 and HitPick online prediction system precision value > 50 were used as potential targets of QHD.

Then, the disease genes related to NAFLD were collected from GeneCards (http://www.gengcards.org/), OMIM (http://omim.org/), and DisGeNET (http://disgenet.org/home/). The key words “NAFLD” were input into GeneCards, OMIM, and DisGeNET databases, respectively, to search for genes related to NAFLD.

Finally, the genes related to NAFLD collected from the three databases were intersected with the predicted potential targets of effective components of QHD. All targets were searched and confirmed by UniProt KB of UniProt (http://www.uniprot.org/).

### 2.3. PPI Network Construction and Mining Analysis

The related target of QHD in the treatment of NAFLD was imported into the STRING (http://string-db.org/) database, where the organism was set to Homo sapiens, and the combination score threshold was 0.7 to obtain the protein-protein interaction relationship. Then, the PPI network of the target protein was constructed and analyzed using the Cytoscape software (3.7.1). The node size in the network was used to reflect the degree size, and the thickness of the edge was used to reflect the comprehensive score size. At the same time, the core submodules of the PPI network were obtained by module mining with the MCODE tool, in which the parameters were set as the default values.

### 2.4. GO Function Enrichment Analysis and KEGG Pathway Annotation Analysis

To further explore the molecular mechanism of QHD in the treatment of NAFLD, DAVID 6.8 (http://david.ncifcrf.gov) was used to analyze the GO function and KEGG pathway enrichment of protein targets; the results were screened using *P* value < 0.05 and FDR < 0.05. According to the results of KEGG pathway enrichment analysis, we established the data file and constructed the target pathway network by using the Cytoscape software (3.7.1).

### 2.5. Docking Verification of Key Target Molecules

In order to verify whether the optimized components of QHD have binding force with the target, the docking verification of the Hub target with a higher degree value in the PPI network was performed using molecular docking technology.

First, SDF format files of 3D structures of these optimized active compounds were downloaded from PubChem (https://pubchem.ncbi.nlm.nih.gov/) database, and protein structure files of key targets were collected from PDB (http://www.rcsb.org/) database according to UniProt ID. Then, PyMOL software (https://pymol.org/) was used to preprocess all small molecules and key targets to remove water molecules and other impurities, all of which were saved as PDB files. Next, the target protein was input into the POCASA 1.1 system (http://altair.sci.hokudai.ac.jp/g6/service/pocasa/) to calculate the optimal binding site (region) of each protein ligand. The best binding site (region) was used as a reference for the docking site. Finally, AutoDock Vina software (http://vina.scripps.edu/) was used for semiflexible molecular docking, and the affinity of all small molecules to their target was calculated and expressed as value (affinity). The smaller the affinity value is, the more stable the interaction between the target protein and the active component is.

## 3. Results

### 3.1. Screening Results of Effective Components of QHD

According to 2.1, 128 components of QHD were screened by the ACD/Labs software, as shown in Supplementary Table 1. Among them, Polygonum cuspidatum has 17 ingredients, turmeric 30, Yinchen 35, gardenia 36, and Tianjihuang 10. Based on SEA, Swiss Target Prediction, and HitPick, 128 effective components were targeted, screened, and sorted, respectively. A total of 1173 pairs of component target interaction relationships were obtained, among which 253 were predicted to have targets.

### 3.2. Target Recognition Results

**Figure 1:**
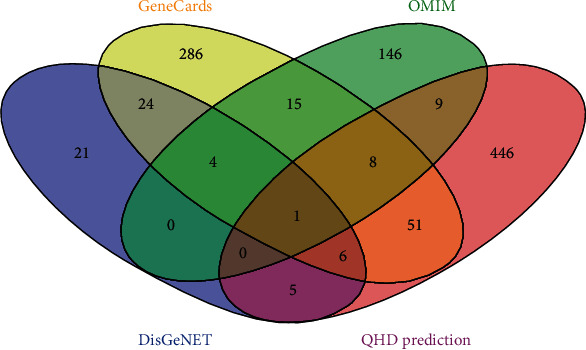
Identification of potential targets of QHD in the treatment of NAFLD.

**Figure 2:**
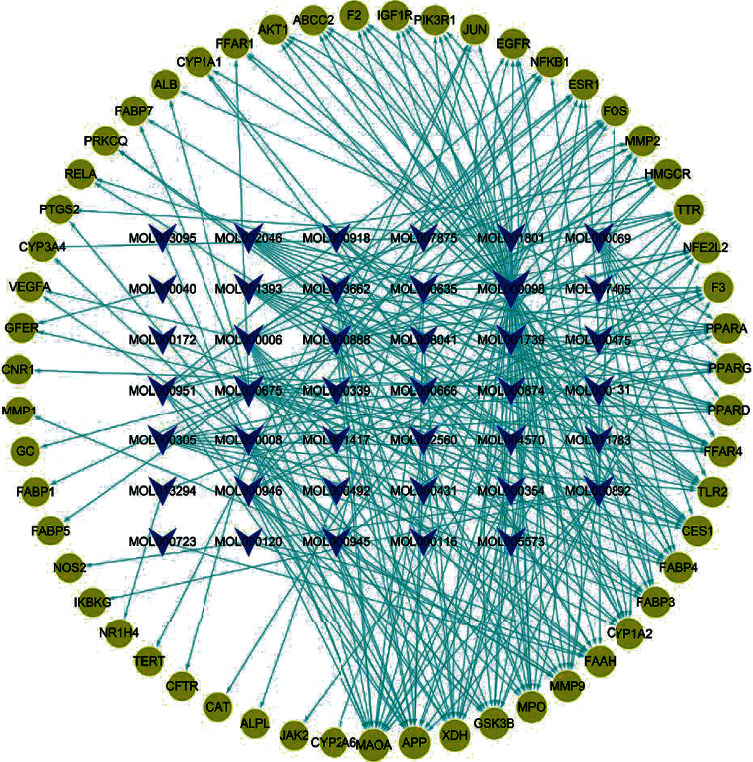
“Compound target” visualization network of QHD. The yellow circle node represents the action target, the blue inverted triangle node represents the compound active component compound, and the green line represents the interaction relationship.

The results showed that 61 genes were found in DisGeNET, 395 in GeneCards, and 183 in OMIM, all of which were related to NAHLD. The target recognition was carried out using the Venny2.1.0 online system, and the results are shown in Figure 1. Finally, 41 main components and 55 targets of QHD in the treatment of NAHLD were obtained, with a total of 551 component target relationships. Forty-one main components and 55 action targets are shown in Supplementary Table 2, and the component target visualization network diagram is shown in Figure 2.

### 3.3. Research on PPI Network Construction and Module Mining

**Figure 3:**
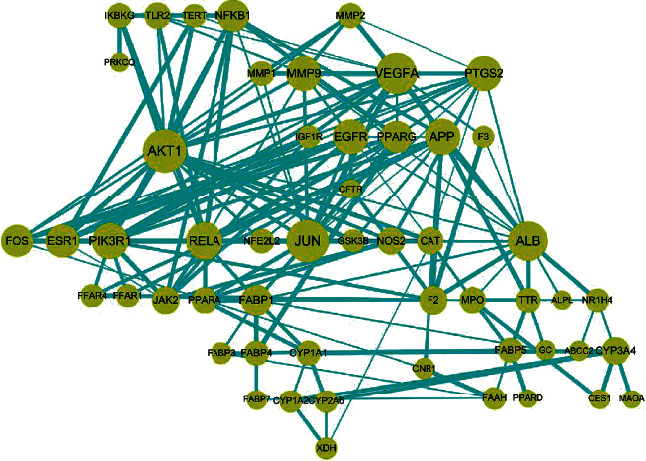
PPI network of related targets of QHD in the treatment of NAFLD.

**Figure 4:**
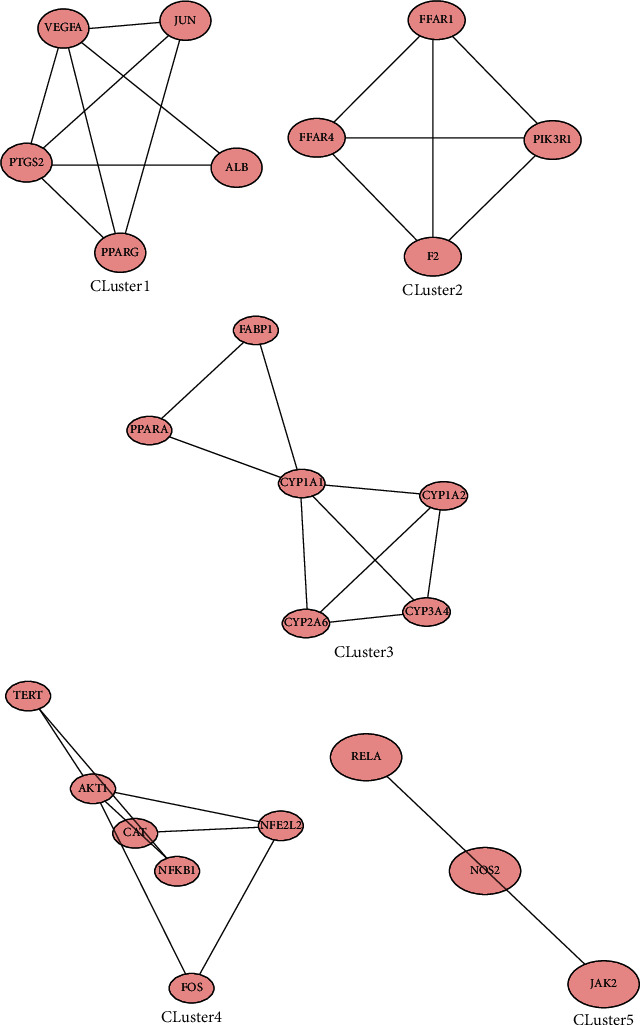
Closely connected submodules in the target network of QHD.

The 55 action targets of QHD in the treatment of NAFLD were imported into the STRING database. According to the method described in 2.3, the protein interaction network was obtained, as shown in Figure 3. Then, based on the MCODE plug-in, the target with a median value and a median value that was not less than the average value was used as the key target of QHD in NAFLD treatment [13], and five submodules with high connectivity were obtained, as shown in Figure 4. The targets in each module and their degree values in the PPI network are shown in Supplementary Table 3.

### 3.4. Results of GO Function Enrichment and KEGG Pathway Annotation

**Figure 5:**
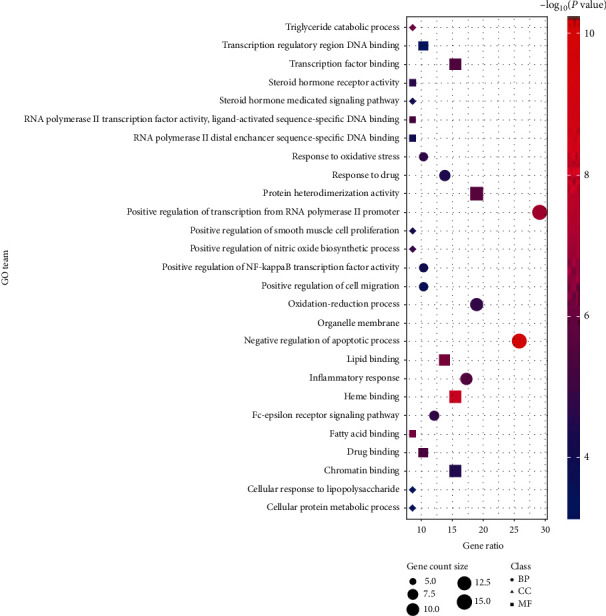
GO function enrichment results of QHD in the treatment of NAFLD. Bubble size is proportional to the number of enriched targets, circle represents BP, triangle represents CC, and square represents MF.

Fifty-five targets were imported into the DAVID 6.8 database, and the GO function (BP, CC, and MF) and KEGG signal pathway were screened according to *P* value < 0.05 and FDR < 0.05. As shown in Figure 5, 15 were found to be related to BP, mainly related to the positive regulation of RNA polymerase II promoter transcription, the oxidation-reduction process, and the negative regulation of apoptosis; 1 was related to CC, mainly involved in organelle membrane; 11 were related to MF, mainly involved in protein heterodimerization activity, lipid binding, transcription factor binding, heme binding, and similar.

**Figure 6:**
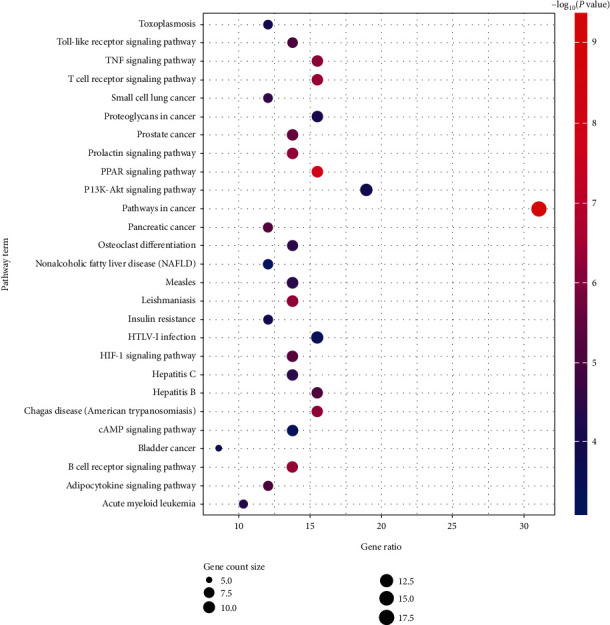
KEGG pathway enrichment results in the treatment of NAFLD.

**Figure 7:**
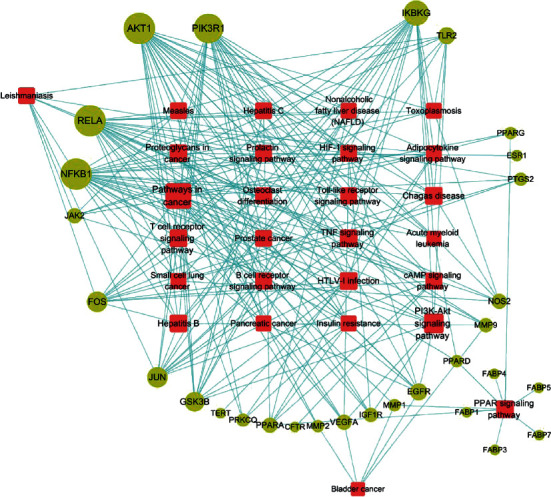
Relationship between target and pathway network. The red square indicates the pathway, and the green circle indicates the target.

GO enrichment analysis showed that NAFLD, as a complex disease, is involved in multiple biological functions. The main chemical components of QHD can adjust these biological functions, thus developing therapeutic effects on nonalcoholic fatty liver. According to the KEGG pathway annotation analysis, as shown in Figure 6, it was found that NAFLD mainly involved cancer pathway (hsa05200), PI3K-AKT pathway (hsa04151), PPAR pathway (hsa03320), and a total of 27 related signaling pathways. Finally, the target-path network of nonalcoholic fatty liver with QHD was constructed according to the target-path relationship as shown in Figure 7.

### 3.5. Molecular Docking Result

**Figure 8:**
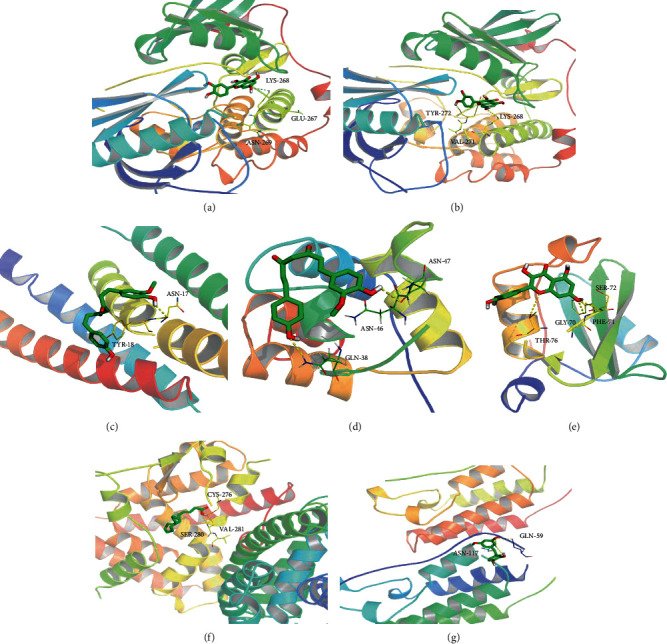
Molecular docking diagram. (a) Represents quercetin and AKT1 docking; (b) represents luteolin and GSK-3*β* docking; (c) represents (1Z,6Z)-1-(4-hydroxy-3-methoxyphenyl)-7-(4-hydroxyphenyl)hepta-1,6-diene-3,5-dione and JUN docking; (d) represents demethoxycurcumin and NFKB1docking; (e) represents quercetin and PIK3R1 docking; (f) represents linoleic acid and PPAR*α* docking; (g) represents cis-3,5,3′,4′-tetrahydroxystilbene and RELA docking.

According to the target degree value in the PPI network, the active ingredients were arranged in degrees. It is generally believed that the higher the degree value, the more disease targets correspond to the components, having a stronger effect [14]. However, we selected targets in the enrichment pathway and considered targets in the disease pathway. Therefore, we have selected targets with degree ≤ 20. The targets of degree ≤ 20, including GSK3B (degree = 4), PPARA (degree = 6), NFKB1 (degree = 10), PIK3R1 (degree = 13), RELA (degree = 14), JUN (degree = 17), and AKT1 (degree = 18), were taken out. The protein crystal files of these 7 targets were downloaded from the PDB database, and the SDF file of the 3D structure of the active compound small molecule was obtained from PubChem. Preprocessing was carried out through PyMOL, and molecular docking was performed using AutoDock Vina. Finally, the affinity values (affinity) of these targets and the small molecules they act on were calculated. The results are shown in Supplementary Table 4. Through molecular docking verification, (1Z,6Z)-1-(4-hydroxy-3-methoxyphenyl)-7-(4-hydroxyphenyl)hepta-1,6-diene-3,5-dione, luteolin, linoleic acid, demethoxycurcumin, quercetin, and cis-3,5,3′,4′tetrahydroxystilbene were found in QHD. These active ingredients had a good affinity with key targets, which reflected that the active ingredients in QHD could treat or improve NAFLD through these Hub targets; the results are shown in Figure 8.

## 4. Discussion

The concepts of “disease” and “syndrome” have been well known in TCM since ancient times. The identification of disease is based on the overall condition of the disease, the pathogenesis of the disease, the syndrome, and the location of the disease. Accordingly, the potential occurrence and development of the disease are understood from a macroperspective [15]. NAFLD belongs to the category of “accumulation,” “fat qi,” “ruffian full,” and “hypochondriac pain” in TCM [16], which has a deep understanding of the etiology, pathogenesis, syndrome differentiation, and drug selection of NAFLD, while the mechanism and pathway of Chinese medicine treatment remain unclear [17]. Network-based pharmacology is beneficial in analyzing the “drug-component-target-disease” interaction network, since it can systematically identify the association between drugs and diseases and reveal the advantages of multimolecule drug synergy [18].

Based on experience from clinical practice, QHD has been used as a representative and effective prescription for NAFLD for a long time [19]. In this study, the effective components and therapeutic targets of QHD were screened through network pharmacology and molecular docking methods, and gene function analysis and KEGG pathway enrichment analysis were performed to clarify the mechanism of QHD in NAFLD treatment. The molecular docking of seven central targets of six key active ingredients in QHD was verified using the AutoDock Vina software, including luteolin, quercetin, demethoxycurcumin, (1Z,6Z)-1-(4-hydroxy-3-methoxyphenyl)-7-(4-hydroxyphenyl)hepta-1,6-diene-3, linoleic acid, and cis-3,5,3′,4′-tetrahydroxystilbene. Quercetin, which is a polyhydroxyflavonoid with multiple biological activities, has high medicinal anti-inflammatory, anticancer, and antioxidative value [20, 21]. Its anticancer mechanism is mainly based on inhibiting the proliferation of liver cancer cells. Luteolin is a natural flavonoid found in many plants that has various pharmacological effects such as anti-inflammatory, antioxidation, antibacterial, nerve protection, anticarcinogenic, and similar [22]. Zhao and Guo [23] and other studies have shown that luteolin could significantly reduce the pathological changes of liver tissue in liver fibrosis rats and had a significant anti-CCl_4_-induced liver fibrosis in rats. Li and Yang [24] found that conjugated linoleic acid can reduce body fat by promoting the burning of fatty acids, inhibiting fat synthesis, and inducing apoptosis of fat cells.

GO enrichment analysis revealed that the key targets of QHD involved biological processes, such as positive regulation of RNA polymerase II promoter transcription, redox processes, and inflammation and negative regulation of apoptosis, protein heterodimers activity, lipid binding, transcription factor binding, heme binding, and similar. Our results revealed biological processes were closely related to cellular DNA replication, proliferation, and signal transduction (such as apoptosis, cytokines, or stress stimulation), thus indicating that QHD had potential therapeutic effects on NAFLD, possibly by affecting biological processes, such as proliferation and apoptosis, inflammation, and oxidative stress. Finally, KEGG analysis revealed that the active ingredients of QHD affected NAFLD, involving cancer pathways, P13K-AKT signaling pathway, PPAR signaling pathway, cAMP signaling pathway, HIF-1 signaling pathway, HTLV-1 infection, and other pathways.

Moreover, previous studies have shown that upregulating the level of miR-373 in liver cells can effectively inhibit AKT signaling and can be used as a strategy for the treatment of fatty liver degeneration [25]. AKT dysfunction leads to glucose and lipid metabolism disorders since it changes the downstream function of kinases or other related signaling molecules that regulate glucose and lipid metabolism. AKT activation is involved not only in glucose metabolism but also in other processes. For example, AKT controls sterol regulatory element-binding proteins (SREBPs) that participate in lipid metabolism. AKT has also been found to regulate the expression of the LDL receptor. Therefore, the AKT signaling pathway is closely related to the regulation of lipid metabolism [26]. Previous studies have shown that the GPS2-PPAR*α* partnership in hepatocytes can coordinate the NAFLD process in mice and humans. It has also been reported that GPS2 acts as an epigenetic genome modifier with selective repressor PPAR*α* hepatocytes and that its inhibitory effect reverses the NASH process to fibrosis [27]. In their study, Zhang et al. [28] found that SIRT1 deacetylation QKI5 affected the expression of RNA-binding protein synthesized in the liver of mice. In addition, it further inhibited triglyceride synthesis by regulating PPAR*α* expression after transcription, thereby limiting the progress of NAFLD in triglyceride synthesis. In the present study, we found that liver biliverdin reductase A (BVRA) inhibits the protective effect of glycogen synthase kinase 3 (GSK3*β*) on hepatic steatosis by enhancing serine 9 phosphorylation. Hinds and colleagues [29] discovered a new BVRA-GSK*β*-PPAR*α* axon that can regulate liver lipid metabolism and provide a unique target for NAFLD treatment. Antrodan (Ant) is a purified glucan extracted from cinnamon *β*-, which has good biological activities, including liver protection, hypolipidemia, antiliver cancer, and anti-inflammatory effects. It has been reported that Ant can effectively alleviate NAFLD through the AMPK/SIRT1/CREB-1C/PPAR*γ* pathway [30]. In our study, overexpression of lysosomal membrane protein (LAMP3) increased AKT phosphorylation and significantly increased the expression of FASN and SCD-1, which were produced by key lipases. In addition, after LY294002 treatment with P13K/AKT pathway inhibitors, LAMP3 overexpression led to a decrease in intracellular lipid accumulation, thus suggesting that LAMP3 may activate the liver cell lipid synthesis by activating the AKT signaling pathway to upregulate key fat formation factor [31]. NAFLD is a related risk factor for hepatocellular carcinoma (HCC). Fatty liver cancer (SH-HCC) is liver cancer characterized by fatty liver, and its occurrence is related to abnormal lipid metabolism. HIF-2*α* activates lipid synthesis by upregulating the PI3K-AKT-mTOR pathway in the hypoxic microenvironment, thus promoting the progression of fatty liver cancer. Therefore, HIF-2*α* can be used as a biomarker for the diagnosis of NAFLD-HCC and a treatment target [32].

Previous pharmacological experiments have found that QHD delayed the lipid deposition in liver cells and also suppressed the inflammatory response and improved liver injury [33]. At the same time, it improved the colon histopathology and ultrastructure in mice with enteritis and NAFLD disease, inhibited intestinal leakage of intestinal toxins, and restored the expression of tight junctions in colon tissue [34].

Huang et al. [35] also confirmed that QHD treated NAFLD by clearing heat and dampness, regulating qi, and activating blood circulation. The results of gene chip and RT-PCR confirmed that QHD could significantly regulate a variety of lipid metabolism pathways, including glyceride metabolism pathway, adipocyte pathway, and PPAR signaling pathway [36]. Furthermore, Qin et al. [37] found that QHD has a significant role in inhibiting liver cancer through the AMPK pathway *in vivo* and *in vitro*.

By using network pharmacology analysis and molecular docking technology, we found that QHD has a potential therapeutic effect on NAFLD, which was consistent with our previous experimental results. Moreover, our results were a reminder that network pharmacology can be used to predict the mechanism of action for a drug in the treatment of the disease.

## 5. Conclusion

The mechanism of action of QHD in the treatment of NAFLD may be related to the following three aspects: QHD controlled lipid metabolism through the AKT signaling pathway. QHD further inhibited the synthesis of triglycerides by regulating PPAR*α* expression after transcription, which in turn limited the synthesis of triglycerides during the occurrence of NAFLD. Liver BVRA inhibited glycogen synthase kinase 3*β* (GSK3*β*) by enhancing the phosphorylation of serine 9 that inhibited its activity and had a protective role against liver fatty degeneration. The mechanism of QHD treatment in NAFLD may be related to the regulation of the BVRA level.

## Data Availability

The data used to support the findings of this study are available from the corresponding author upon request.

## Supplementary Materials

Supplementary 1 Table 1: active ingredients of QHD.

Supplementary 2 Table 2: 41 main active components of QHD.

Supplementary 3 Table 3: target data of submodule.

Supplementary 4 Table 4: docking results of some core components and key target molecules of QHD.
