# Supplementary material for: Research on the Mechanism of Qushi Huayu Decoction in the Intervention of Nonalcoholic Fatty Liver Disease Based on Network Pharmacology and Molecular Docking Technology
Source: Biomed Res Int. 2020 Nov 4;2020:1704960. doi: 10.1155/2020/1704960 (PMC7658690; doi:10.1155/2020/1704960)
Supplement: Supplementary 1 — Table 1: active ingredients of QHD. [file 1704960.f1.pdf]

Table 1 Active ingredients of QHD

Herb	Molecule name	InChlKey	Pubchem Cid	Canonical Smiles	Molecular Formula
HZ	4'-Methoxyacetophenone	NTPLXRHDUXRPNE-UHFFFAOYSA-N	7476	CC(=O)C1=CC=C(C=C1)OC	C9H10O2
HZ	Ambrettolide	NVIPUOMWGQAOIT-RQOWECAXSA-N	5365703	C1CCCCOC(=O)CCCCCC=CCCC1	C16H28O2
HZ	(1R,12R)-17-Methoxy-5,7,11,19-tetraoxapentacyclo[10.8.0.02,10.04,8.013,18]icosa-2,4(8),9,13(18),14,16-hexaen-16-ol	UXAJJVDCDOFKCY-BONVTDFDSA-N	44559428	COC1=C(C=CC2=C1OCC3C2OC4=CC5=C(C=C34)OCO5)O	C17H14O6
HZ	7-Hydroxy-2,5-dimethyl-4H-1-benzopyran-4-one	CRNGFKXWIYTEPH-UHFFFAOYSA-N	5316891	CC1=CC(=CC2=C1C(=O)C=C(O2)C)O	C11H10O3
HZ	Physovenine	LXTKNVLLWOLCOV-JSGCOSHPSA-N	442113	CC12CCOC1N(C3=C2C=C(C=C3)OC(=O)NC)C	C14H18N2O3
HZ	(4Ar,5R,6aR,6aS,6bR,8aS,9S,10S,12aR,14bR)-9-formyl-5,10-dihydroxy-2,2,6a,6b,9,12a-hexamethyl-1,3,4,5,6,6a,7,8,8a,10,11,12,13,14b-tetradecahydropicene-4a-carboxylic acid	MQUFAARYGOUYEV-XCBGTDNPSA-N	99646993	CC1(CCC2(C(C1)C3=CCC4C5(CCC(C(C5CCC4(C3(CC2O)C)C)(C)C=O)O)C)C(=O)O)C	C30H46O5
HZ	Torachrysone 8-O-Glucoside	GHKWPHRULCFTBB-CZNQJBLBSA-N	11972479	CC1=CC2=CC(=CC(=C2C(=C1C(=O)C)O)OC3C(C(C(C(O3)CO)O)O)O)OC	C20H24O9
HZ	Torachrysone	BIJOPUWEMBBDEG-UHFFFAOYSA-N	5321977	CC1=CC2=CC(=CC(=C2C(=C1C(=O)C)O)O)OC	C14H14O4
HZ	Emodin anthrone	LAJSXCAVRQXZIO-UHFFFAOYSA-N	122635	CC1=CC2=C(C(=C1)O)C(=O)C3=C(C2)C=C(C=C3O)O	C15H12O4
HZ	Coumarin	ZYGHJZDHTFUPRJ-UHFFFAOYSA-N	323	C1=CC=C2C(=C1)C=CC(=O)O2	C9H6O2
HZ	cis-3,5,3',4'-Tetrahydroxy	CDRPUGZCR	6603962	C1=CC(=C(C=C	C14H12O4

	stilbene	XZLFL-UPHRSURJSA-N		1C=CC2=CC(=CC(=C2)O)O)O)O	
HZ	Cianidanol	PFTAWBLQPZVEMU-DZGCQCFKSA-N	9064	C1C(C(OC2=CC(=CC(=C21)O)O)C3=CC(=C(C=C3)O)O)O	C15H14O6
HZ	3,5-Dimethyl-4-methoxybenzoic acid	WXVQURJGDUNJCS-UHFFFAOYSA-N	88944	CC1=CC(=CC(=C1OC)C)C(=O)O	C10H12O3
HZ	Luteolin	IQPNAANSBPBGFQ-UHFFFAOYSA-N	5280445	C1=CC(=C(C=C1C2=CC(=O)C3=C(C=C(C=C3O2)O)O)O)O	C15H10O6
HZ	2-Methoxyhydroquinone	LAQYHRQFABOIFD-UHFFFAOYSA-N	69988	COC1=C(C=CC(=C1)O)O	C7H8O3
HZ	Apigenin	KZNIFHPLKGYRTM-UHFFFAOYSA-N	5280443	C1=CC(=CC=C1C2=CC(=O)C3=C(C=C(C=C3O2)O)O)O	C15H10O5
HZ	Quercetin	REFJWTPEDVJJIY-UHFFFAOYSA-N	5280343	C1=CC(=C(C=C1C2=C(C(=O)C3=C(C=C(C=C3O2)O)O)O)O)O	C15H10O7
JH	Bisabolol	RGZSQWQPBWRIAQ-LSDHHAIUSA-N	1549992	CC1=CCC(CC1)C(C)(CCC=C(C)C)O	C15H26O
JH	D-Limonene	XMGQYMWWDOXHJM-JTQLQIEISA-N	440917	CC1=CCC(CC1)C(=C)C	C10H16
JH	(+)-Borneol	DTGKSKDOIYIVQL-WEDXCCLWSA-N	6552009	CC1(C2CCC1(C(C2)O)C)C	C10H18O
JH	(-)-alpha-Curcumene	VMYXUZSZMNBRCN-CQSZACIVSA-N	442360	CC1=CC=C(C=C1)C(C)CCC=C(C)C	C15H22
JH	Anethole	RUVINXPYWBROJD-ONEGZZNKSA-N	637563	CC=CC1=CC=C(C=C1)OC	C10H12O
JH	(+)-alpha-Pinene	GRWFGVWFFZKLTI-RKDXNWHRSA-N	82227	CC1=CCC2CC1C2(C)C	C10H16
JH	alpha-Cedrene	IRAQOCYXUMOFCW-OSF	6431015	CC1CCC2C13CC=C(C(C3)C2(C	C15H24

		YFWSMSA-N		)C)C	
JH	(1Z,6Z)-1-(4-Hydroxy-3-methoxyphenyl)-7-(4-hydroxyphenyl)hepta-1,6-diene-3,5-dione	HJTVQHVGMGKONQ-AVWDGMTFSA-N	92855592	COC1=C(C=CC(=C1)C=CC(=O)CC(=O)C=CC2=CC=C(C=C2)O)O	C20H18O5
JH	(+)-alpha-Curcumene	VMYXUZSZMNBRCN-AWEZNQCLSA-N	3083834	CC1=CC=C(C=C1)C(C)CCC=C(C)C	C15H22
JH	(1Z,6Z)-1,7-Bis(4-hydroxy-3-methoxyphenyl)hepta-1,6-diene-3,5-dione	VFLDPWHFBUODDF-VHOZIDCHSA-N	6604598	COC1=C(C=CC(=C1)C=CC(=O)CC(=O)C=CC2=CC(=C(C=C2)O)OC)O	C21H20O6
JH	Curcumenone	HUZJLWLCLJEXEL-NFAWXSAZSA-N	153845	CC(=C1CC2C(C2(CC1=O)C)CCC(=O)C)C	C15H22O2
JH	4(5H)-Benzofuranone, 6-ethenyl-6,7-dihydro-3,6-dimethyl-5-(1-methylethenyl)-, trans-	ZVMJXSJCBLRAPD-ZFWWWQNUSA-N	3081930	CC1=COC2=C1C(=O)C(C(C2)(C)C=C)C(=C)C	C15H18O2
JH	(+)-Curcumenol	ISFMXVMWEWLJGJ-NZBPQXDJSA-N	167812	CC1CCC2C13CC(=C(C)C)C(O3)(C=C2C)O	C15H22O2
JH	Curcumol	QRMPRVXWPCLVNI-YYFQZIEXSA-N	14240392	CC1CCC2C13CC(C(O3)(CC2=C)O)C(C)C	C15H24O2
JH	(+)-beta-Pinene	WTARULDDTDQWMU-RKDXNWHRSA-N	10290825	CC1(C2CCC(=C)C1C2)C	C10H16
JH	alpha-Zingiberene	KKOXKGNSUHTUBV-CABCVRRESA-N	11127403	CC1=CCC(C=C1)C(C)CCC=C(C)C	C15H24
JH	2-Hepten-4-one, 2-methyl-6-(4-methylphenyl)-, (6R)-	NAAJVHHFAXWBOK-CYBMUJFWSA-N	11736067	CC1=CC=C(C=C1)C(C)CC(=O)C=C(C)C	C15H20O
JH	(1Z,6E)-1,7-Bis(4-hydroxyphenyl)hepta-1,6-diene-3,5-dione	PREBVFJICNPEKM-JTAXDKCCSA-N	45934475	C1=CC(=CC=C1C=CC(=O)CC(=O)C=CC2=CC=C(C=C2)O)O	C19H16O4
JH	Demethoxycurcumin	HJTVQHVGMGKONQ-LUZURFALSA-N	5469424	COC1=C(C=CC(=C1)C=CC(=O)CC(=O)C=CC2=	C20H18O5

				CC=C(C=C2)O)O	
JH	Isoprocurcumenol	ITIGZFMSPAFZAE-WHOFXGATSA-N	14543198	CC(=C1CC2C(CCC2(C)O)C(=C)CC1=O)C	C15H22O2
JH	(4Z,6Z)-5-Hydroxy-1,7-bis(4-hydroxy-3-methoxyphenyl)hepta-4,6-dien-3-one	BWHPKBOLJFNCPW-FZFQPXCJSA-N	78350454	COC1=C(C=CC(=C1)CCC(=O)C=C(C=CC2=CC(=C(C=C2)O)OC)O)O	C21H22O6
JH	(3S,3As,8aS)-3-hydroxy-3,8-dimethyl-5-propan-2-ylidene-2,3a,4,8a-tetrahydro-1H-azulen-6-one	RHBOHEXDGUVIIY-ZLDLUXBVSA-N	10263440	CC1=CC(=O)C(=C(C)C)CC2C1CCC2(C)O	C15H22O2
JH	(6S)-2-Methyl-6-[(1S)-4-methylidenecyclohex-2-en-1-yl]hept-2-en-4-one	JIJQKFPGBBEJNF-KBPBESRZSA-N	14287405	CC(CC(=O)C=C(C)C)C1CCC(=C)C=C1	C15H22O
JH	(3R,6E,10S)-6,10-Dimethyl-3-propan-2-ylcyclodec-6-ene-1,4-dione	KDPFMRXIVDLQKX-OAIDTJHVSA-N	10466651	CC1CCC=C(CC(=O)C(CC1=O)C(C)C)C	C15H24O2
JH	(3R,3As,8S,8aR)-3,8-dihydroxy-3,8-dimethyl-5-propan-2-ylidene-1,2,3a,4,7,8a-hexahydroazulen-6-one	TXIKNNOOLCGADE-OSRDXIQISA-N	24834047	CC(=C1CC2C(CCC2(C)O)C(CC1=O)(C)O)C	C15H24O3
JH	(3S,3Ar,8aS)-3,3a-dihydroxy-3,8-dimethyl-5-propan-2-ylidene-1,2,4,8a-tetrahydroazulen-6-one	MBUWIGIPGMJVMN-AEGPPILISA-N	14633012	CC1=CC(=O)C(=C(C)C)CC2(C1CCC2(C)O)O	C15H22O3
JH	Procurcumenol	RHBOHEXDGUVIIY-WHOFXGATSA-N	189061	CC1=CC(=O)C(=C(C)C)CC2C1CCC2(C)O	C15H22O2
JH	Bisacurone	QJOWFYQIUZMPRY-NEBZKDRISA-N	14287397	CC(CC(=O)C=C(C)C)C1CC(C(C=C1)(C)O)O	C15H24O3
JH	(+)-Endo-beta-bergamotene	DGZBGCMPRYFWFF-KKUMJFAQSA-N	12300073	CC(=CCCC1(C2CCC(=C)C1C2)C)C	C15H24
JH	2,3-Dimethyl-2,3-diphenylbutane	HGTUJZTUQFXBIH-UHFFFAOYSA-N	74681	CC(C)(C1=CC=CC=C1)C(C)(C)C2=CC=CC=C2	C18H22
YC	(-)-alpha-Terpineol	WUOACPNHFRMFPN-SECBINFHSA-N	443162	CC1=CCC(CC1)C(C)(C)O	C10H18O
YC	(-)-beta-Pinene	WTARULDDT	440967	CC1(C2CCC(=C	C10H16

		DQWMU-IUCAKERBSA-N		)C1C2)C	
YC	Furfural	HYBBIBNJHNGZAN-UHFFFAOYSA-N	7362	C1=COC(=C1)C=O	C5H4O2
YC	Salicylic acid	YGSDEFSMJLZEOE-UHFFFAOYSA-N	338	C1=CC=C(C(=C1)C(=O)O)O	C7H6O3
YC	Scoparone	GUAFOGOEJLSQBT-UHFFFAOYSA-N	8417	COC1=C(C=C2C(=C1)C=CC(=O)O2)OC	C11H10O4
YC	Methyleugenol	ZYEMGPIYFIJGTP-UHFFFAOYSA-N	7127	COC1=C(C=C(C=C1)CC=C)OC	C11H14O2
YC	Eugenol	RRAFCDWBNXTKKO-UHFFFAOYSA-N	3314	COC1=C(C=CC(=C1)CC=C)O	C10H12O2
YC	4'-Hydroxyacetophenone	TXFPEBPIARQUIG-UHFFFAOYSA-N	7469	CC(=O)C1=CC=C(C=C1)O	C8H8O2
YC	Isoscopoletin	SYTYLPHCLSSCOJ-UHFFFAOYSA-N	69894	COC1=C(C=C2C=CC(=O)OC2=C1)O	C10H8O4
YC	Isorhamnetin	IZQSVPBOUDKVDZ-UHFFFAOYSA-N	5281654	COC1=C(C=CC(=C1)C2=C(C(=O)C3=C(C=C(C=C3O2)O)O)O)O	C16H12O7
YC	Scopoletin	RODXRVNMMDRFIK-UHFFFAOYSA-N	5280460	COC1=C(C=C2C(=C1)C=CC(=O)O2)O	C10H8O4
YC	Arcapillin	IZWKTABKAJGBFW-UHFFFAOYSA-N	158311	COC1=C(C=C(C(=C1)C2=CC(=O)C3=C(C(=C(C=C3O2)OC)OC)O)O)O	C18H16O8
YC	Butyraldehyde	ZTQSAGDEMFDKMZ-UHFFFAOYSA-N	261	CCCC=O	C4H8O
YC	Capillarin	KUJLPCYCQICVRM-UHFFFAOYSA-N	3083811	CC#CCC1=CC2=CC=CC=C2C(=O)O1	C13H10O2
YC	Genkwanin	JPMYFOBNRRGFNO-UHFF	5281617	COC1=CC(=C2C(=C1)OC(=CC	C16H12O5

		FAOYSA-N		2=O)C3=CC=C(C=C3)O)O	
YC	Vanillin	MWOOGOJBHIARFG-UHFFFAOYSA-N	1183	COC1=C(C=CC(=C1)C=O)O	C8H8O3
YC	Artepillin C	KABCFARPAMSXCC-JXMROGBWSA-N	5472440	CC(=CCC1=CC(=CC(=C1O)CC=C(C)C)C=CC(=O)O)C	C19H24O3
YC	Cirsilineol	VKOSQMWSWLZQPA-UHFFFAOYSA-N	162464	COC1=C(C=CC(=C1)C2=CC(=O)C3=C(C(=C(C=C3O2)OC)OC)O)O	C18H16O7
YC	Eupalitin	KWMAWXWUGIEVDG-UHFFFAOYSA-N	5748611	COC1=C(C(=C2C(=C1)OC(=C(C2=O)O)C3=CC=C(C=C3)O)O)OC	C17H14O7
YC	Eupatolitin	WYKWHSPRHPZRCR-UHFFFAOYSA-N	5317291	COC1=C(C(=C2C(=C1)OC(=C(C2=O)O)C3=CC(=C(C=C3)O)O)O)OC	C17H14O8
YC	3,4',5-Trihydroxy-7-methoxyflavanone	LZLGHWHSUZVUFZ-JKSUJKDBSA-N	181132	COC1=CC(=C2C(=C1)OC(C(C2=O)O)C3=CC=C(C=C3)O)O	C16H14O6
YC	Capillarisin	NTKNGUAZSFAKEE-UHFFFAOYSA-N	5281342	COC1=C(C2=C(C=C1O)OC(=CC2=O)OC3=CC=C(C=C3)O)O	C16H12O7
YC	5,7-Dihydroxy-6-methoxy-2-(4-methoxyphenoxy)chromen-4-one	ZFSSSVBLDHAXFV-UHFFFAOYSA-N	5319540	COC1=CC=C(C=C1)OC2=CC(=O)C3=C(O2)C=C(C(=C3O)OC)O	C17H14O7
YC	6-Demethoxycapillarisin	UBSCDKPKWHYZNX-UHFFFAOYSA-N	5316511	C1=CC(=CC=C1O)OC2=CC(=O)C3=C(C=C(C=C3O2)O)O	C15H10O6
YC	Capillartemisin A	HEFPIIHDRLNTDN-JBFPSKHUSA-N	6439717	CC(=CCC1=C(C(=CC(=C1)C=CC(=O)O)CC=C(	C19H24O4

				C)CO)O)C	
YC	Capillartemisin B	HEFPIIHDRLNTDN-ZCVOOGJLSA-N	6439716	CC(=CCC1=C(C(=CC(=C1)C=CC(=O)O)CC=C(C)CO)O)C	C19H24O4
YC	Hexa-1,3-diynylbenzene	BRCIUCONXSZSKN-UHFFFAOYSA-N	5320055	CCC#CC#CC1=CC=CC=C1	C12H10
YC	Capillene	WXQYRBLGGSLJHA-UHFFFAOYSA-N	3083613	CC#CC#CCC1=CC=CC=C1	C12H10
YC	1,3-Pentadiynylbenzene	ACPMYIIORVILBG-UHFFFAOYSA-N	10983572	CC#CC#CC1=CC=CC=C1	C11H8
YC	5-Phenyl-1,3-pentadiyne	ZZGANZXITREHOP-UHFFFAOYSA-N	587245	C#CC#CCC1=CC=CC=C1	C11H8
YC	Capillin	RAZOKRUZEQERLH-UHFFFAOYSA-N	10321	CC#CC#CC(=O)C1=CC=CC=C1	C12H8O
YC	beta-Elemene	OPFTUNCRGUEPRZ-QLFBSQMISA-N	6918391	CC(=C)C1CCC(C(C1)C(=C)C)(C)C=C	C15H24
YC	(-)-3-Carene	BQOFWKZOCNGFEC-DTWKUNHWSA-N	442461	CC1=CCC2C(C1)C2(C)C	C10H16
YC	2-Nonanone	VKCYHJWLYTUGCC-UHFFFAOYSA-N	13187	CCCCCCCC(=O)C	C9H18O
YC	Quercetin	REFJWTPEDVJJIY-UHFFFAOYSA-N	5280343	C1=CC(=C(C=C1C2=C(C(=O)C3=C(C=C(C=C3O2)O)O)O)O)O	C15H10O7
ZZ	Genipin	AZKVWQKMDGGDSV-BCMRRPTOSA-N	442424	COC(=O)C1=COC(C2C1CC=C2CO)O	C11H14O5
ZZ	Quercetin	REFJWTPEDVJJIY-UHFFFAOYSA-N	5280343	C1=CC(=C(C=C1C2=C(C(=O)C3=C(C=C(C=C3O2)O)O)O)O)O	C15H10O7
ZZ	D-Limonene	XMGQYMWWDOXHJM-JTQLQIEISA-N	440917	CC1=CCC(CC1)C(=C)C	C10H16

ZZ	Nonanal	GYHFUZHODSMOHU-UHFFFAOYSA-N	31289	CCCCCCCCC=O	C9H18O
ZZ	2-Decenal	MMFCJPPRCYDLLZ-CMDGGOBGSA-N	5283345	CCCCCCCC=CC=O	C10H18O
ZZ	Octyl acetate	YLYBTZIQSIBWLI-UHFFFAOYSA-N	8164	CCCCCCCCOC(=O)C	C10H20O2
ZZ	Linoleic acid	OYHQOLUKZRVURQ-HZJYTTRNSA-N	5280450	CCCCCC=CCC=CCCCCCCCC(=O)O	C18H32O2
ZZ	Lauric acid	POULHZVOKOAJMA-UHFFFAOYSA-N	3893	CCCCCCCCCCCC(=O)O	C12H24O2
ZZ	Episyringaresinol	KOWMJRJXZMEZLD-GKHNXXNSSA-N	12309694	COC1=CC(=CC(=C1O)OC)C2C3COC(C3CO2)C4=CC(=C(C(=C4)OC)O)OC	C22H26O8
ZZ	Eucarvone	QNGQIURXCUHNAT-UHFFFAOYSA-N	136330	CC1=CC=CC(CC1=O)(C)C	C10H14O
ZZ	Hexanal	JARKCYVAAOWBJS-UHFFFAOYSA-N	6184	CCCCCC=O	C6H12O
ZZ	2-Pentylfuran	YVBAUDVGOFCUSG-UHFFFAOYSA-N	19602	CCCCCC1=CC=CO1	C9H14O
ZZ	Oleic acid	ZQPPMHVWECSIRJ-KTKRTIGZSA-N	445639	CCCCCCCCC=CCCCCCCCC(=O)O	C18H34O2
ZZ	2,4-Decadienal	JZQKTMZYLHNFPL-BLHCBFLLSA-N	5283349	CCCCCC=CC=CC=O	C10H16O
ZZ	Paeonol	UILPJVPSNHJFIK-UHFFFAOYSA-N	11092	CC(=O)C1=C(C=C(C=C1)OC)O	C9H10O3
ZZ	Germacr-1(10)-ene-5,8-dione	KDPFMRXIVDLQKX-NHFJXKHHSA-N	6441391	CC1CCC=C(CC(=O)C(CC1=O)C(C)C)C	C15H24O2
ZZ	(3S)-3,7-Dimethylocta-1,6-dien-3-ol	CDOSHBSSFJOMGT-SNVBAGLBSA-N	67179	CC(=CCCC(C)(C=C)O)C	C10H18O

ZZ	(3S,6E)-Nerolidol	FQTLCLSUCSAZDY-ATGUSINASA-N	5281525	CC(=CCCC(=CCCC(C)(C=C)O)C)C	C15H26O
ZZ	(-)-beta-Elemene	OPFTUNCRGUEPRZ-ZNMIVQPWSA-N	9859094	CC(=C)C1CCC(C(C1)C(=C)C)(C)C=C	C15H24
ZZ	Myristic acid	TUNFSRHWOTWDNC-UHFFFAOYSA-N	11005	CCCCCCCCCCCCCC(=O)O	C14H28O2
ZZ	2-Octenal	LVBXEMGDVWVTGY-VOTSOKGWSA-N	5283324	CCCCCC=CC=O	C8H14O
ZZ	(6S,10R)-6,10,14-Trimethylpentadecan-2-one	WHWDWIHXSPCOKZ-SJORKVTESA-N	1810796	CC(C)CCCC(C)CCCC(C)CCCC(=O)C	C18H36O
ZZ	[(4S,7S,8R,11S)-8-Hydroxy-2-oxo-3,9-dioxatricyclo[5.3.1.04,11]undeca-1(10),5-dien-6-yl]methyl acetate	NGGKNZCFVQZFGK-MIZYBKAJSA-N	71766875	CC(=O)OCC1=CC2C3C1C(OC=C3C(=O)O2)O	C12H12O6
ZZ	Methyl (1R,4aS,5R,7aS)-1,5-dihydroxy-7-(hydroxymethyl)-1,4a,5,7a-tetrahydrocyclopenta[c]pyran-4-carboxylate	QMBSLGFBNUKGRJ-SDNRWEOFSA-N	71577496	COC(=O)C1=COC(C2C1C(C=C2CO)O)O	C11H14O6
ZZ	Palmitoleic acid	SECPZKHBENQXJG-FPLPWBNLSA-N	445638	CCCCCCC=CCCCCCCCC(=O)O	C16H30O2
ZZ	Isophorone	HJOVHMDZYOCNQW-UHFFFAOYSA-N	6544	CC1=CC(=O)CC(C1)(C)C	C9H14O
ZZ	Isoimperatorin	IGWDEVSBEKYORK-UHFFFAOYSA-N	68081	CC(=CCOC1=C2C=CC(=O)OC2=CC3=C1C=CO3)C	C16H14O4
ZZ	Hexanoic acid	FUZZWVXGSFPDMH-UHFFFAOYSA-N	8892	CCCCCC(=O)O	C6H12O2
ZZ	Chrysin	RTIXKCRFFJGDFG-UHFFFAOYSA-N	5281607	C1=CC=C(C=C1)C2=CC(=O)C3=C(C=C(C=C3O2)O)O	C15H10O4
ZZ	Corymbosin	FLCVGMVLN	10970376	COC1=CC(=C2	C19H18O7

		HYJAW-UHFF FAOYSA-N		C(=C1)OC(=CC 2=O)C3=CC(=C (C(=C3)OC)OC) OC)O	
ZZ	7,4'-Dihydroxyflavone	LCAWNFIFM LXZPQ-UHFF FAOYSA-N	5282073	C1=CC(=CC=C1 C2=CC(=O)C3= C(O2)C=C(C=C 3)O)O	C15H10O4
ZZ	Benzyl acetate	QUKGYYKBI LRGFE-UHFF FAOYSA-N	8785	CC(=O)OCC1= CC=CC=C1	C9H10O2
ZZ	N-Phenyl-1-naphthylamin e	XQVWYOYU ZDUNRW-UH FFFAOYSA-N	7013	C1=CC=C(C=C1)NC2=CC=CC3 =CC=CC=C32	C16H13N
ZZ	Methyl benzoate	QPJVMBTYP HYUOC-UHF FFAOYSA-N	7150	COC(=O)C1=C C=CC=C1	C8H8O2
ZZ	Methyl (1R,4aS,5R,7S,7aS)-1,5,7 -trihydroxy-7-methyl-4a,5 ,6,7a-tetrahydro-1H-cyclo penta[c]pyran-4-carboxyl ate	GGZSQSUXB PYCHQ-BEBV ASNESA-N	25026345	CC1(CC(C2C1C (OC=C2C(=O)O C)O)O)O	C11H16O6
ZZ	2-Ethyl-3-propylacrolein	PYLMCYQHB RSDND-SOFG YWHQSA-N	5354264	CCCC=C(CC)C =O	C8H14O
TJH	Vanillic acid	WKOLLVMJN QIZCI-UHFFF AOYSA-N	8468	COC1=C(C=CC(=C1)C(=O)O)O	C8H8O4
TJH	Bisabolol	RGZSQWQPB WRIAQ-LSDH HAIUSA-N	1549992	CC1=CCC(CC1) C(C)(CCC=C(C) C)O	C15H26O
TJH	Dibutyl phthalate	DOIRQSBPFJ WKBE-UHFFF AOYSA-N	3026	CCCCOC(=O)C 1=CC=CC=C1C(=O)OCCCC	C16H22O4
TJH	Palmitic acid	IPCSVZSSVZ VIGE-UHFFF AOYSA-N	985	CCCCCCCCCC CCCCCC(=O)O	C16H32O2
TJH	Sarothralin	CTSAWQOSU XMFIZ-UHFF FAOYSA-N	354448	CC(C)C(=O)C1= C(C(=C(C(C1=O)(C)C)O)CC2=C (C(=C(C=C2O) OCC=C(C)C)C(=O)C3=CC=CC	C31H34O8

				=C3)O)O	
TJH	Saroaspidin A	QNAVIUVOJCTDPT-UHFFFAOYSA-N	3082648	CC1=C(C(=C(C(=C1O)C(=O)C(C)C)O)CC2=C(C(C(=O)C(=C2O)C(=O)C(C)C)(C)C)O)O	C24H30O8
TJH	Isoamyl laurate	FVKRIDSRWFEQME-UHFFFAOYSA-N	61386	CCCCCCCCCCCC(=O)OCCC(C)C	C17H34O2
TJH	Dimethyl sulfone	HHVIBTZHLRERCL-UHFFFAOYSA-N	6213	CS(=O)(=O)C	C2H6O2S
TJH	3',4',5,7-Tetramethoxyflavone	CLXVBVLQKLQNRQ-UHFFFAOYSA-N	631170	COC1=C(C=C(C=C1)C2=CC(=O)C3=C(O2)C=C(C=C3OC)OC)OC	C19H18O6
TJH	Quercetin	REFJWTPEDVJJIY-UHFFFAOYSA-N	5280343	C1=CC(=C(C=C1C2=C(C(=O)C3=C(C=C(C=C3O2)O)O)O)O)O	C15H10O7
