# Supplementary material for: Research on the Mechanism of Qushi Huayu Decoction in the Intervention of Nonalcoholic Fatty Liver Disease Based on Network Pharmacology and Molecular Docking Technology
Source: Biomed Res Int. 2020 Nov 4;2020:1704960. doi: 10.1155/2020/1704960 (PMC7658690; doi:10.1155/2020/1704960)
Supplement: Supplementary 2 — Table 2: 41 main active components of QHD. [file 1704960.f2.pdf]

Table 2 41 main active components of QHD

Herb	TCMSP ID	PubChem Name	PubChem CID	Molecular Formula
HZ	MOL013294	(4Ar,5R,6aR,6aS,6bR,8aS,9S,10S,12aR,14bR)-9-formyl-5,10-dihydroxy-2,2,6a,6b,9,12a-hexamethyl-1,3,4,5,6,6a,7,8,8a,10,11,12,13,14b-tetradecahydropicene-4a-carboxylic acid	99646993	C30H46O5
HZ	MOL000431	Coumarin	323	C9H6O2
HZ	MOL004570	cis-3,5,3',4'-Tetrahydroxystilbene	6603962	C14H12O4
HZ	MOL000492	Cianidanol	9064	C15H14O6
HZ	MOL011783	4'-Methoxyacetophenone	7476	C9H10O2
HZ	MOL000006	Luteolin	5280445	C15H10O6
HZ	MOL000008	Apigenin	5280443	C15H10O5
JH	MOL000892	(1Z,6Z)-1,7-Bis(4-hydroxy-3-methoxyphenyl)hepta-1,6-diene-3,5-dione	6604598	C21H20O6
JH	MOL000946	Demethoxycurcumin	5469424	C20H18O5
JH	MOL000945	(1Z,6E)-1,7-Bis(4-hydroxyphenyl)hepta-1,6-diene-3,5-dione	45934475	C19H16O4
JH	MOL000888	(1Z,6Z)-1-(4-Hydroxy-3-methoxyphenyl)-7-(4-hydroxyphenyl)hepta-1,6-diene-3,5-dione	92855592	C20H18O5
JH	MOL000951	(4Z,6Z)-5-Hydroxy-1,7-bis(4-hydroxy-3-methoxyphenyl)hepta-4,6-dien-3-one	78350454	C21H22O6
JH	MOL000475	Anethole	637563	C10H12O
YC	MOL000918	2-Nonanone	13187	C9H18O
YC	MOL007405	Cirsilineol	162464	C18H16O7
YC	MOL001801	Salicylic acid	338	C7H6O3
YC	MOL000040	Scopoletin	5280460	C10H8O4
YC	MOL000635	Vanillin	1183	C8H8O3
YC	MOL008041	Eupatolitin	5317291	C17H14O8
YC	MOL000172	Furfural	7362	C5H4O2
YC	MOL005573	Genkwanin	5281617	C16H12O5
YC	MOL000354	Isorhamnetin	5281654	C16H12O7
YC	MOL000339	Isoscopoletin	69894	C10H8O4
ZZ	MOL000305	Lauric acid	3893	C12H24O2
ZZ	MOL000131	Linoleic acid	5280450	C18H32O2
ZZ	MOL000723	2,4-Decadienal	5283349	C10H16O
ZZ	MOL000120	2-Decenal	5283345	C10H18O
ZZ	MOL000666	Hexanal	6184	C6H12O
ZZ	MOL001417	2-Octenal	5283324	C8H14O
ZZ	MOL003662	7,4'-Dihydroxyflavone	5282073	C15H10O4
ZZ	MOL002560	Chrysin	5281607	C15H10O4
ZZ	MOL001393	Myristic acid	11005	C14H28O2
ZZ	MOL002046	Hexanoic acid	8892	C6H12O2
ZZ	MOL000116	Nonanal	31289	C9H18O

ZZ	MOL000675	Oleic acid	445639	C18H34O2
ZZ	MOL000874	Paeonol	11092	C9H10O3
ZZ	MOL001739	Palmitoleic acid	445638	C16H30O2
TJH	MOL000098	Quercetin	5280343	C15H10O7
TJH	MOL000069	Palmitic acid	985	C16H32O2
TJH	MOL007875	Dimethyl sulfone	6213	C2H6O2S
