# Supplementary material for: Research on the Mechanism of Qushi Huayu Decoction in the Intervention of Nonalcoholic Fatty Liver Disease Based on Network Pharmacology and Molecular Docking Technology
Source: Biomed Res Int. 2020 Nov 4;2020:1704960. doi: 10.1155/2020/1704960 (PMC7658690; doi:10.1155/2020/1704960)
Supplement: Supplementary 3 — Table 3: target data of submodule. [file 1704960.f3.pdf]

Table 3. Target data of sub module

Cluster	Score	Gene（Degree）
Cluster1	4	PTGS2(12),PPARG(10),VEGFA(16), ALB(15),JUN(17)
Cluster2	4	FFAR4(4),F2(8),PIK3R1(13),FFAR1(4)
Cluster3	3.6	PPARA(6),CYP1A2(4),CYP3A4(7),CYP2A6(4),CYP1A1(5),FABP1(9)
Cluster4	3.2	TERT(4),AKT1(18),NFKB1(10),CAT(7),FOS(10),NFE2L2(5)
Cluster5	3	RELA(14),JAK2(7),NOS2(7)
