# Supplementary material for: Research on the Mechanism of Qushi Huayu Decoction in the Intervention of Nonalcoholic Fatty Liver Disease Based on Network Pharmacology and Molecular Docking Technology
Source: Biomed Res Int. 2020 Nov 4;2020:1704960. doi: 10.1155/2020/1704960 (PMC7658690; doi:10.1155/2020/1704960)
Supplement: Supplementary 4 — Table 4: docking results of some core components and key target molecules of QHD. [file 1704960.f4.pdf]

Table 4. Docking results of some core components and key target molecules of QHD

Ligand	Receptor	Affinity(kcal/mol)
(1Z,6Z)-1-(4-Hydroxy-3-methoxyphenyl)-7-(4-hydroxyphenyl) hepta-1,6-diene-3,5-dione	JUN	-6.0
Luteolin	GSK-3β	-8.7
Linoleic acid	PPARα	-5.8
Demethoxycurcumin	NFKB1	-5.9
Quercetin	AKT1	-9.8
Quercetin	PIK3R1	-6.2
cis-3,5,3',4'-Tetrahydroxystilbene	RELA	-7
